# Overexpression of rice aquaporin *OsPIP1;2* improves yield by enhancing mesophyll CO_2_ conductance and phloem sucrose transport

**DOI:** 10.1093/jxb/ery386

**Published:** 2018-12-11

**Authors:** Feiyun Xu, Ke Wang, Wei Yuan, Weifeng Xu, Shuang Liu, Herbert J Kronzucker, Guanglei Chen, Rui Miao, Maoxing Zhang, Ming Ding, Liang Xiao, Lei Kai, Jianhua Zhang, Yiyong Zhu

**Affiliations:** 1 Jiangsu Collaborative Innovation Center for Solid Organic Waste Resource Utilization, College of Resources and Environmental Science, Nanjing Agricultural University, Nanjing, China; 2 College of Life Sciences and Joint International Research Laboratory of Water and Nutrient in Crops, Fujian Agriculture and Forestry University, Fuzhou, China; 3 School of Agriculture and Food, Faculty of Veterinary and Agricultural Sciences, The University of Melbourne, VIC, Australia; 4 The Key Laboratory of Biotechnology for Medicinal Plants of Jiangsu Province, Jiangsu Key Laboratory of Phylogenomics and Comparative Genomics, School of Life Sciences, Jiangsu Normal University, Xuzhou, China; 5 Department of Biology, Hong Kong Baptist University, and the State Key Laboratory of Agrobiotechnology, Chinese University of Hong Kong, Hong Kong, China

**Keywords:** Aquaporin, elevated CO_2_, grain yield, mesophyll conductance, rice, sucrose

## Abstract

Aquaporins are involved in CO_2_ transport from the leaf intercellular air space to the chloroplast, which contributes to CO_2_ assimilation. However, the mechanism of CO_2_ transport by rice (*Oryza sativa* L.) aquaporins is unknown. Here, we investigated the function of the aquaporin OsPIP1;2 in CO_2_ diffusion-associated photosynthesis and phloem sucrose transport. Moreover, the grain yield of rice lines overexpressing *OsPIP1;2* was determined. OsPIP1;2 was localized to the plasma membrane and the relative expression of *OsPIP1;2* was approximately 5-fold higher in leaves in the presence of an elevated CO_2_ concentration. Overexpression of *OsPIP1;2* increased mesophyll conductance by approximately 150% compared with wild-type (WT) rice. The *OsPIP1;2*-overexpressing lines had higher biomass than the WT, possibly due to increased phloem sucrose transport. In addition, the grain yield of *OsPIP1;2*-overexpressing lines was approximately 25% higher than that of the WT in three-season field experiments, due to the increased numbers of effective tillers and spikelets per panicle. Our results suggest that *OsPIP1;2* modulates rice growth and grain yield by facilitating leaf CO_2_ diffusion, which increases both the net CO_2_ assimilation rate and sucrose transport.

## Teaser

*OsPIP1;2* overexpression enhances rice growth and grain yield by facilitating leaf CO_2_ diffusion, which increases the net CO_2_ assimilation rate and phloem sucrose transport.

## Introduction

The atmospheric CO_2_ concentration ([CO_2_]) has increased significantly from 318 to >400 ppm since 1959 (Meinshausen *et al.*, 2011). The rate of increase in atmospheric [CO_2_] may accelerate (Tripati *et al.*, 2009), and is predicted to reach 550–700 ppm by 2050 (Meinshausen *et al.*, 2011). Therefore, the effect of elevated [CO_2_] on crop production has been intensively investigated in recent decades (Kimball, 2016). Rice (*Oryza sativa* L.) is a major staple food crop for almost half the global population (Kurai *et al.*, 2011). The yield of various rice cultivars is reportedly improved by an elevated [CO_2_], as indicated by increased growth, tiller number, and leaf area (Kimball, 2016; Hasegawa *et al.*, 2013). An elevated [CO_2_] had a positive effect on leaf gas exchange and net photosynthetic rate (Norby *et al.*, 2016), and thus is important for plant growth and development.

In general, CO_2_ entering chloroplasts must pass through leaf stomata, plasma membranes, cytoplasm, and chloroplast membranes; these steps are collectively reflected by stomatal conductance (*g*_s_) and leaf mesophyll conductance to CO_2_ (*g*_m_) (Evans and Loreto, 2000; Evans *et al.*, 2009). In rice, *g*_s_ is a limiting factor in photosynthesis (Kusumi *et al.*, 2012), and enhanced *g*_s_ increased biomass in Arabidopsis (Wang *et al.*, 2014). Although *g*_m_ was long considered to be constant, it is now known to vary according to environmental conditions (Montpied *et al.*, 2009; Singh *et al.*, 2014). Flexas *et al.* (2008) reported that *g*_m_ is an important determinant of the photosynthetic rate, indicating that CO_2_ diffusion from the leaf intercellular air space to the chloroplast is a limiting factor in photosynthesis. Aquaporin NtAQP1 from tobacco leaf facilitates CO_2_ transport across the plasma membrane *in vivo*, which in turn modulates membrane permeability to CO_2_ and mesophyll conductance (Uehlein *et al.*, 2003). In addition, NtAQP1 is located in the inner chloroplast membrane, and reduced expression of NtAQP1 resulted in a 20% decrease in CO_2_ conductance (Uehlein *et al.*, 2008). By contrast, overexpression of *NtAQP1* in tobacco significantly increased *g*_m_. In Arabidopsis, T-DNA insertion of *atpip1;2* reduced leaf CO_2_ conductivity, indicating that *AtPIP1;2* facilitates CO_2_ transport (Heckwolf *et al.*, 2011). Moreover, overexpression of the barley aquaporin *HvPIP2;1* in rice plants resulted in an increased *g*_m_ value (Hanba *et al.*, 2004).

OsPIP1;2 is a plasma membrane intrinsic protein (PIP) localized to cellular plasma membranes (Lian *et al.*, 2006). Sakurai *et al.* (2005) reported that rice plants possess 33 aquaporin-encoding genes and stop-flow spectrophotometry analysis revealed that OsPIP2;4 and OsPIP2;5, but not OsPIP1;2, have high water-channel activity. In addition, OsPIP1 members are localized mainly to mesophyll cells (Sakurai *et al.*, 2005). According to the sequence homology of *PIP* genes in various plant species, the role of *OsPIP1;2* may be related to CO_2_ diffusion in rice plants; however, the evidence is sparse. In this study, we evaluated the function of OsPIP1;2 in CO_2_ permeability using *OsPIP1;2*-overexpressing (OE) rice lines under ambient and elevated [CO_2_] by determining the biomass, photosynthesis-related physiological parameters, and phloem sucrose-transport rate. Moreover, the contribution of *OsPIP1;2* to rice yield was analysed in a field experiment. We aimed to determine the function of *OsPIP1;2* in rice plants and its potential for agriculture.

## Materials and methods

### Plant growth conditions

Rice seeds were sterilized as described by Zhu *et al.* (2009) for hydroponic experiments. After 10 d, seedlings of OE lines and the wild type (WT) were transplanted into 7-litre plastic containers. Rice plants were grown in a growth chamber (Saifu DRX-680E-DG-CO_2_, Ningbo, China) under a light intensity of 300 μmol m^−2^ s^−1^ at shoot height, a relative humidity of *ca* 70%, and a 14 h light (26 °C)/10 h dark (22 °C) photoperiod. Each experiment was randomized and involved three replicates of five plants each at ambient [CO_2_] (400 μmol mol^−1^) and elevated [CO_2_] (800 μmol mol^−1^). The nutrient solution contained: 1.25 mM NH_4_NO_3_, 0.3 mM K_2_SO_4_, 0.3 mM NaH_2_PO_4_, 1 mM CaCl_2_, 1 mM MgSO_4_, 9 μM MnCl_2_, 0.39 μM Na_2_MoO_4_, 20 μM H_3_BO_4_, 0.77 μM ZnSO_4_, 0.32 μM CuSO_4_, and 20 μM EDTA-Fe. Nutrient solution was exchanged every 3 d and its pH was maintained at 5.5. The expression level of *OsPIP1;2* was determined in cv. Nipponbare at the tillering, booting, flowering, and grain-filling stages grown in soil pots from June to October 2016; three replications were used.

### Homology modeling and sequence alignment

We performed homology modeling using the workspace at the Swiss-Model website (http://swissmodel.expasy.org/). The X-ray crystal structures of SoPIP2;1 (Protein Data Bank [PDB] codes: 2D5F and 1Z98) served as templates for homology modeling (Törnroth-Horsefield *et al.*, 2006). The amino acid sequence of aquaporin OsPIP1;2 was aligned using Jalview software version 1.6 (http://www.jalview.org/).

### Construction of *OsPIP1;2*-transgenic rice plants

For β-glucuronidase (GUS) expression analysis, the *OsPIP1;2* (Os04g47220) promoter (1977 bp) was amplified from rice (*Oryza sativa* L. cv Nipponbare) genomic DNA using the primers listed in Supplementary Table S1 at *JXB* online. The fragment was ligated into the *Sal*I/*Kpn*I sites of the vector pS1aG3 to replace the cauliflower mosaic virus 35S promoter (Tang *et al.*, 2012). The open reading frame (ORF) sequence of *OsPIP1;2* was amplified using the primers listed in Supplementary Table S1. Generation of the *OsPIP1;2*-OE vector was described by Patron *et al.* (2015).

### Histochemical localization of GUS expression

Histochemical analysis was performed as described previously (Ai *et al.*, 2009). Leaves and roots of rice plants were collected in triplicate after 3 weeks. Inflorescences were selected prior to flowering, and seeds were selected 30 d after pollination. Tissues were immersed in GUS reaction mix for 30 min and subsequently incubated at 37 °C for 2 h. GUS-stained tissues were visualized using an Olympus BX51T stereomicroscope equipped with a color charge-coupled device camera.

### Transient expression of *OsPIP1;2* and fluorescence microscopy

The ORF of *OsPIP1;2* without the stop codon was cloned into the C-terminus of the pCAMBIA (GFP) vector at *Hin*dIII/*Pst*I sites. Next, the *35S::OsPIP1;2::GFP* expression vector was transferred into rice protoplasts using polyethylene glycol-mediated transformation. Rice protoplasts were obtained from etiolated seedlings and transfected as described previously (Jia *et al.*, 2011). OsMCA1 was used as a plasma membrane localization marker (Takamitsu *et al.*, 2012). A confocal laser scanning microscope (LSM410; Carl Zeiss, Oberkochen, Germany) was used to obtain fluorescence images.

### Reverse transcription-polymerase chain reaction and real-time quantitative PCR

To investigate the expression pattern of *OsPIP1;2*, samples were taken from rice plants at different growth stages (Yamaji *et al.*, 2013). To determine the expression level of *OsPIP1;2* in leaf and the effect of CO_2_ on its expression, rice seedlings (2 weeks old) were exposed to ambient [CO_2_] (400 μmol mol^−1^) or elevated [CO_2_] (800 μmol mol^−1^) for 1 week in a growth chamber (Saifu DRX-680E-DG-CO_2_).

Total RNA was isolated using TRIzol reagent (Invitrogen, Carlsbad, CA, USA). *OsPIP1;2* and *OsActin* were subjected to reverse transcription-polymerase chain reaction (RT-PCR) and real-time quantitative RT-PCR using the primers in Supplementary Tables S2 and S3 and the protocol of Zeng *et al.* (2012).

### Field experiments

Transgenic T4- and T6-generation rice plants were cultivated in plots at the Nanjing Agricultural University experimental site from June to October in 2016 and 2017. T5-generation rice plants were grown in plots of the experimental site of Sanya Nanjing Agricultural University (tropical climate) from December 2016 to April 2017. The soil at the experimental site contained 29.42 g kg^−1^ organic carbon, 25.78 mg kg^−1^ Olsen-P, and 140 mg kg^−1^ exchangeable potassium. The pH of the soil was 6.4. Nitrogen (urea, 200 kg ha^−1^), phosphorus (P_2_O_5_, 90 kg ha^−1^), and potassium (K_2_O, 150 kg ha^−1^) were applied for the present experiment. Nitrogen fertilizer was split into basal dressing, panicle initiation, and initial spikelet differentiation during the growing season at a ratio of 50:30:20. Phosphorus and potassium fertilizer was applied only as a basal dressing prior to transplanting. The field experiment was set up in triplicate randomized plots of 2 × 2.5 m. Four randomly selected samples were taken from plants in each plot at the flowering and harvest stages, and the data for 12 plants were calculated. Effective tiller number, spikelets per panicle, and grain yield were calculated at the harvest stage.

### Gas exchange and chlorophyll fluorescence measurements

The LI-6400 system (LI-COR, Lincoln, NE, USA) was used for measuring gas exchange and chlorophyll fluorescence in plants grown in the growth chamber and the field. The temperature of the leaf chamber was maintained at 25 °C, with a photosynthetically active radiation (PPFD) of 1500 μmol m^−2^ s^−1^. The ambient CO_2_ concentration was adjusted to 400 μmol m^−2^ s^−1^. The relative humidity in the leaf chamber was maintained at 50–60%. After equilibration to a steady state, the gas-exchange parameters, steady-state fluorescence (*F*_s_), and maximum fluorescence (*F*_m_′) were recorded. For *A*_net_–*C*_i_ curves, the leaf was adjusted to 1500 μmol m^−2^ s^−1^ PPFD, 400 μmol CO_2_ mol^−1^, and 25 °C. The relative humidity in the leaf chamber was maintained at 50–60%. Before the measurement, three rice leaves were heated until they did not have chlorophyll fluorescence. They were used to correct the leakage of the measured *A*_net_–*C*_i_ curves (Flexas *et al.*, 2007). Measurements were started when the net rate of CO_2_ assimilation became constant at 400 μmol CO_2_ mol^−1^ under saturating light (1500 μmol m^−2^ s^−1^). The ambient [CO_2_] was increased stepwise from 50 to 1000 μmol CO_2_ mol^−1^ at intervals of 20 min. After dark adaptation for 30 min, light-response curves were recorded in steps of more than 20 min duration (stable statue) at nine PPFDs (0, 50, 100, 150, 200, 400, 600, 1000, and 1500 μmol m^−2^ s^−1^). The measurement conditions were as described above, except that the light intensity was increased stepwise. Newly and fully expanded leaves were selected for measurement at 09.00–15.00 h daily.

Efficiency of photosystem II electron transport (Φ_PSII_) was calculated as Φ_PSII_=1−*F*_s_/*F*_m_′. The electron transport rate (*J*) was calculated as follows:

$$J\;=\Phi_{\text{PSII}}\times \text{PPFD}\times \text{αβ}$$ (1)

Where α is leaf absorption and β is the proportion of quanta between PSI and PSII. The product αβ was obtained by varying the PPFDs under non-photorespiratory conditions in the presence of less than 2% O_2_ (Valentini *et al.*, 1995). The *g*_m_ and chloroplast CO_2_ concentration (*C*_c_) were calculated by the variable *J* method (Harley *et al.*, 1992) as follows:

$$g_{\text{m}}=\frac{A}{C_{\text{i}}-\frac{\Gamma *\left(J+8\left(A+R_{\text{d}}\right)\right)}{J-4\left(A+R_{\text{d}}\right)}}$$ (2)

$$C_{\text{c}}=C_{\text{i}}-\frac{A}{g_{\text{m}}}\;\;$$ (3)

where *A* is net rate of CO_2_ assimilation, *C*_i_ is the substomatal CO_2_ concentration and Г* is the CO_2_ compensation point in the absence of respiration. In the present study, a Г* value of 40 μmol mol^−1^, typical for rice plants, was used based on Franks and Farquhar (2001) and Giuliani *et al.* (2013). Daytime respiration rate (*R*_d_) was calculated as the intercept of the linear regression of the photosynthetic rate against PPFD×Φ_PSII_/4 using light-response curve data (Yin *et al.*, 2009). The *R*_d_ values of transgenic plants are listed in Supplementary Fig. S1.

A curve-fitting method (Ethier and Livingston, 2004; Ethier *et al.*, 2006) was used to calculate the maximum Rubisco activity (*V*_cmax_) and maximum electron transport rate (*J*_max_) as described by Sharkey *et al.* (2007).

The leaf net rate of CO_2_ assimilation (*A*_net_) in the paddy field was measured on August 10–11 (flowering stage), August 20–21 (middle grain-filling stage), and September 4–5 (end grain-filling stage). The temperature of the leaf chamber was maintained at 25 °C, with a PPFD of 1500 μmol m^−2^ s^−1^. The relative humidity in the leaf chamber was maintained at 50–60%. Four random samples from three plots per group (*n*=12) were selected for measurement. Newly and fully expanded leaves were selected for measurement at 09.00–11.00 h daily.

### Measurement of leaf chlorophyll content, stomatal density, stomatal size, relative water content, dry mass per unit area and water use efficiency

Chlorophyll was extracted with ethanol and quantified using a SpectraMax M5 spectrometer (Molecular Devices, Sunnyvale, CA, USA) (Sartory and Grobbelaar, 1984; Xiong *et al.*, 2015). The stomatal density (*n*=15) and size (*n*=30) of leaf adaxial and abaxial surfaces were measured as described by Wang *et al.* (2011, 2014). Briefly, three fully expanded leaves of rice plants were selected, and the stomatal density and size were determined in five randomly selected microphotographs of the adaxial or abaxial surface of the lamina. Leaf number of three plants was measured. In the field experiment, after the leaf net rate of CO_2_ assimilation was measured for plants, the leaf chlorophyll content was measured using a chlorophyll meter (SPAD 502 Plus; Spectrum Technologies, Japan). The leaf relative water content and dry mass per unit area were measured according to Heckwolf *et al.* (2011). Water us efficiency (WUE) was calculated as the ratio between net rate of CO_2_ assimilation and transpiration rate.

### Measurement of leaf N content

Leaf samples were harvested, heated at 105 °C for 30 min, and dried at 80 °C for 3 d. The leaf samples were digested with 18.4 M H_2_SO_4_ at 260–270 °C and their N contents were determined using an Auto Analyzer 3 digital colorimeter (Bran + Luebbe GmbH, Germany).

### Measurement of phloem sucrose transport and sucrose content in tissues

Phloem exudates were collected as described previously (King and Zeevaart, 1974). Petioles were cut in 10 M EDTA (pH 6.0), transferred to a cup containing 1.0 ml EDTA, and incubated in the dark for 1 h for exudation.

The sucrose contents of rice-plant tissues were measured in five biological replicates according to Stitt *et al.* (1989). Samples (0.1 g) were extracted three times with 4 ml of 80% v/v ethanol for 20 min at 80 °C. Next, the samples were incubated for 10 min in boiling water. The sucrose content was measured using a SpectraMax M5 spectrometer.

### Statistical analysis

The data were subjected to analysis of variance and Duncan’s multiple-range test using SPSS 18.0 software (SPSS Inc., Chicago, IL, USA). A value of *P*<0.05 was considered indicative of statistical significance.

## Results

### Localization and expression pattern of *OsPIP1;2* in rice plants

**Fig. 1.**
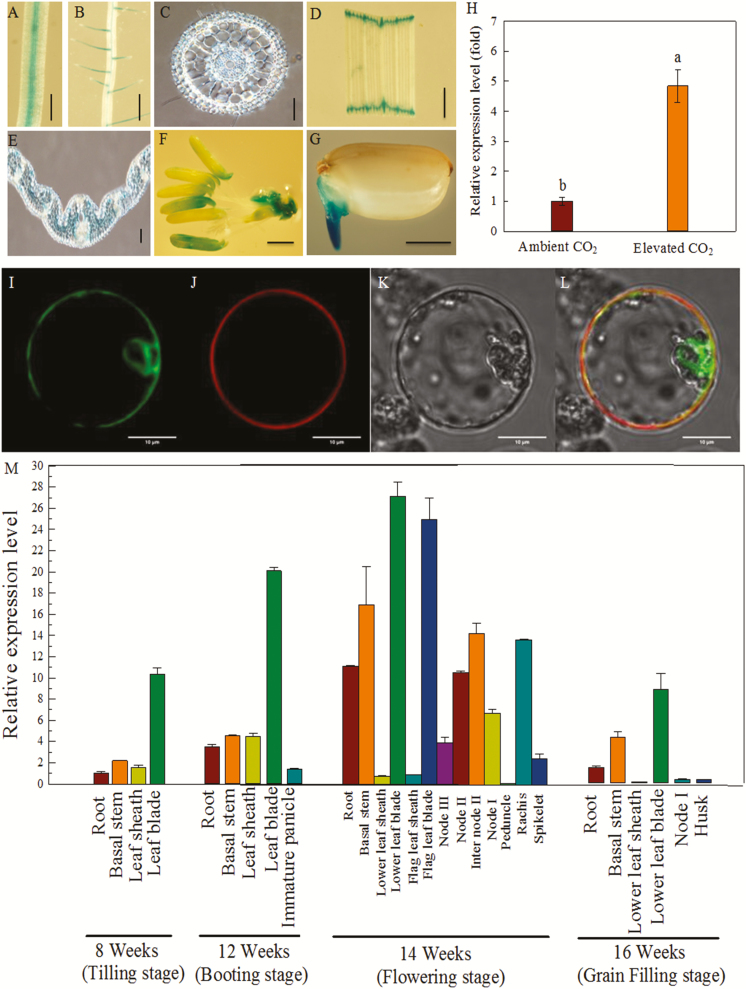
*OsPIP1;2* expression pattern and subcellular localization. (A–G) Rice transformed with *OsPIP1;2* promoter::GUS. GUS activity in the root (A), lateral root elongation zone (B), cross-section of the root tip (C), leaf blade (D), cross-section of the leaf blade (E), floret (F), and germinating seeds (G). Scale bars: 100 μm (C, E) and 1 mm (A, B, D, F, G). (H) Response of *OsPIP1;2* expression to CO_2_ in leaf blade by quantitative real-time PCR. The internal reference gene was *OsActin*. Values are means ±SD (*n*=3); different letters indicate significant differences (*P*<0.05). (I–L) Subcellular localization of OsPIP1;2 in rice protoplasts. Scale bars: 10 μm. (I) Fluorescence signal of 35S::OsPIP1;2-GFP. (J) fluorescence signal of OsMCA1 (Takamitsu *et al.*, 2012). (K) bright-field image. (L) merged image. (M) Expression levels of *OsPIP1;2* in the indicated tissues and at the indicated growth stages of the wild type (WT) (cv. Nipponbare) grown in soil, as determined by quantitative real-time PCR with *OsActin* as an internal reference. Values are means ±SD (*n*=3).

The amino acid sequences of OsPIP1;2 and other aquaporins (NtAQP1, AtPIP1;2, HvPIP2;1, and HvPIP2;3) are highly conserved. A homology model of OsPIP1;2 was generated using the crystal structure of SoPIP2;1 (PDB codes: 2D5F and 1Z98) as a template (Supplementary Fig. S2). To analyse the expression of *OsPIP1;2* in rice tissue, a 1977 bp fragment immediately upstream of the translation start site of *OsPIP1;2* was used for GUS reporter (Fig. 1A–G). *OsPIP1;2* was expressed in the roots of rice plants (Fig. 1A–C). GUS activity was high in the leaf, panicle, and embryonic primary tissue (Fig. 1D, F, G). In the cross-section of the leaf blade, GUS activity was detected in mesophyll cells (Fig. 1E).

By real-time quantitative RT-PCR, the relative expression level of *OsPIP1;2* in the rice leaf blade was 5-fold higher in the presence of an elevated [CO_2_] (Fig. 1H). The expression of *OsPIP1;2* was next investigated in different organs and growth stages (Fig. 1M). *OsPIP1;2* showed the highest expression in the leaf blade at all growth stages. In addition, the expression of *OsPIP1;2* in the leaf blade was highest at the flowering stage. *OsPIP1;2* was also expressed in other organs at various levels (Fig. 1M).

To determine the subcellular localization of OsPIP1;2, an OsPIP1;2–GFP fusion was expressed in mature rice protoplasts isolated from culms of rice seedlings grown in the dark. The GFP fluorescence signal was detected at the plasma membrane (Fig. 1I–L), indicating co-localization with the plasma membrane marker Ca^2+^-permeable mechanosensitive channel OsMAC1 (Takamitsu *et al.*, 2012). Therefore, OsPIP1;2 is localized to the plasma membrane.

### Gas-exchange parameters of rice plants

**Fig. 2.**
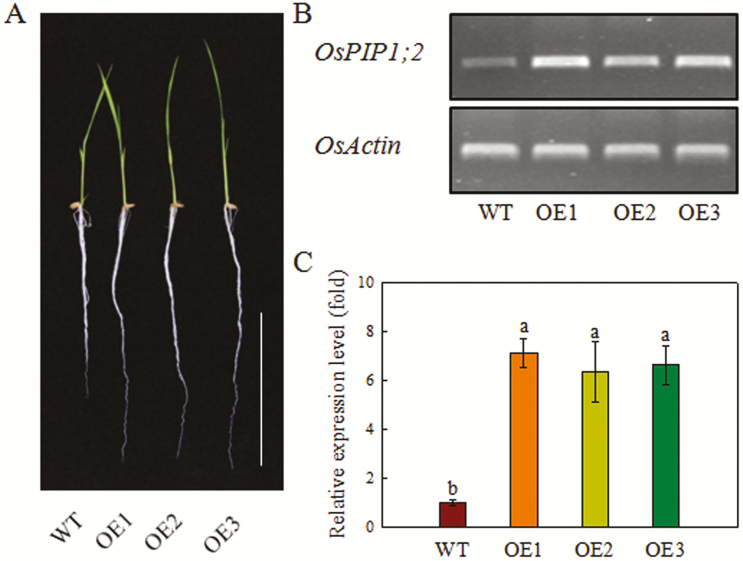
Expression level of *OsPIP1;2* in selected rice transgenic lines. (A) Phenotype of 7-day-old transgenic lines [overexpressing (OE) 1, OE2, and OE3]. Scale bar: 10 cm. (B, C) Expression level of *OsPIP1;2* in the OE lines by RT-PCR (B) and real-time quantitative PCR (C) with *OsActin* as an internal reference. Values are means ±SD (*n*=3); different letters indicate significant differences (*P*<0.05).

To characterize the physiological function of OsPIP1;2 in rice plants, OE lines were constructed. The relative expression level of *OsPIP1;2* was significantly 6.3–7.1-fold higher in the OE lines than in the WT (Fig. 2).

**Fig. 3.**
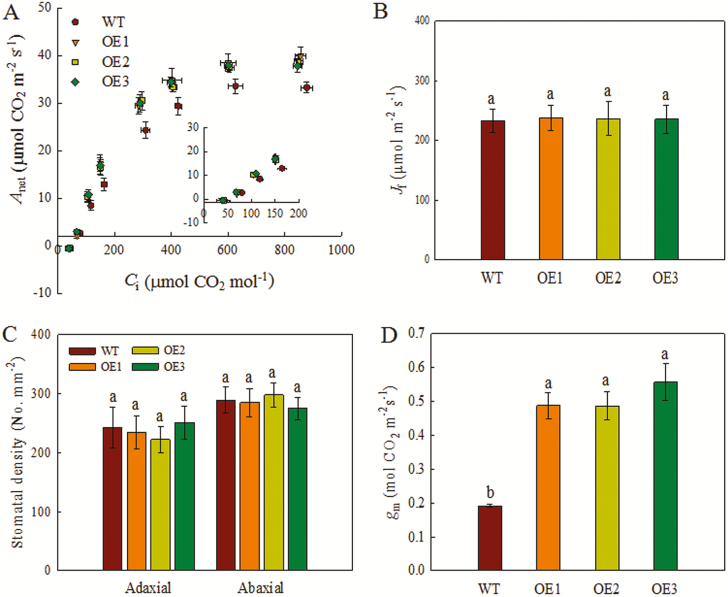
Net rate of CO_2_ assimilation response curves and parameters. Rice plants were grown under ambient [CO_2_] in growth chambers for 4 weeks. (A) The response of the net rate of CO_2_ assimilation to the substomatal CO_2_ concentration (*C*_i_) (*n*=5). (B) Electron transport rate (*J*_f_) (*n*=5). (C) Stomatal density on the adaxial and abaxial surfaces of leaves (*n*=15). (D) Mesophyll CO_2_ conductance (*g*_m_) of rice plants (*n*=5). All values are means ±SD; different letters indicate significant differences (*P*<0.05).

We next examined the net rate of CO_2_ assimilation (*A*_net_) at different substomatal CO_2_ concentrations (*C*_i_) and PPFDs using a gas-exchange system (Fig. 3; Supplementary Fig. S3). The *A*_net_ values revealed that the response of the OE lines to a low PPFD (<200 μmol m^−2^ s^−1^) was similar to that of the WT (Supplementary Fig. S3). However, at a PPFD of 1500 μmol m^−2^ s^−1^, the *A*_net_ of the OE lines was 17–19% higher than that of the WT (Supplementary Fig. S3). Under these conditions, *A*_net_ was light-saturated and limited by the carboxylation rate. The light-use efficiency of the OE lines was higher than that of the WT under light-saturated conditions. The *A*_net_ of the OE lines was also higher than that of the WT at a *C*_i_ of >150 μmol CO_2_ mol^−1^ (Fig. 3A). The maximum *A*_net_ of the OE lines was ~37 μmol CO_2_ m^−2^ s^−1^, compared with ~31 μmol m^−2^ s^−1^ in the WT. Therefore, the OE lines had greater CO_2_ for photosynthesis. Based on the chlorophyll fluorescence and gas exchange data, the leaf *g*_m_ of the OE lines increased by approximately 150% that of the WT (Fig. 3D). The *g*_s_ values of the OE lines were 26–38% higher than that of the WT (Supplementary Fig. S4). By contrast, water-use efficiency did not differ significantly between the OE lines and the WT (Supplementary Fig. S5). Further, there was no significant difference between the OE lines and the WT in the electron transport rate (*J*_f_) and maximum electron transport rate (*J*_max_) (Fig. 3B; Table 1). The *C*_c_ and maximum Rubisco activity (*V*_cmax_) of the OE lines were 25–30% and 28–33%, respectively, higher than those of the WT (Table 1).

**Table 1.** Net rate of CO_2_ assimilation parameters of rice plants under ambient [CO_2_]

	Anet (μmol CO2 m−2 s−1)	Ci (μmol CO_2_ mol−1)	Cc (μmol CO2 mol−1)	Vcmax (μmol CO2 m−2 s−1)	Jmax (μmol photons m−2 s−1)
WT	28.05 ± 1.3b	305 ± 9a	158 ± 16b	55.9 ± 4.8b	242 ± 11a
OE1	33.43 ± 1.6a	267 ± 18b	198 ± 15a	71.8 ± 4.6a	251 ± 9a
OE2	32.95 ± 1.5a	272 ± 16b	204 ± 8a	74.1 ± 5.8a	246 ± 17a
OE3	33.19 ± 1.1a	265 ± 14b	205 ± 10a	72.4 ± 6.5a	248 ± 26a

Rice plants were grown under ambient [CO_2_] in the chamber for 4 weeks. Values are means ±SD (*n*=5). Different letters indicate significant differences at the *P*<0.05 level in rice plants. *A*_net_, net rate of CO_2_ assimilation; *C*_c_, chloroplastic CO_2_ concentration; *C*_i_, substomata CO_2_ concentration; *J*_max_, maximum electron transport rate; *V*_cmax_, maximum Rubisco activity.

In the field experiment, the *A*_net_ values of the OE lines were 9–15%, 13–42%, and 19–34% higher than those of the WT at the flowering, middle-filling, and end-filling stages, respectively. In addition, the *A*_net_ values of the OE lines and the WT decreased from the flowering stage to the end of the grain-filling stage. There was no significant difference in chlorophyll content at any growth stage between the OE lines and the WT (Supplementary Fig. S6).

The OE lines and the WT did not display significant differences in chlorophyll content, number of leaves per plant, relative water content, leaf dry mass per unit area (Supplementary Table S4), or stomatal density and size on the adaxial or abaxial surface (Fig. 3C; Supplementary Table S5). In addition, the leaf N content was similar between the OE lines and the WT (Supplementary Fig. S7).

### Response of *OsPIP1;2* to ambient and elevated [CO_2_]

**Fig. 4.**
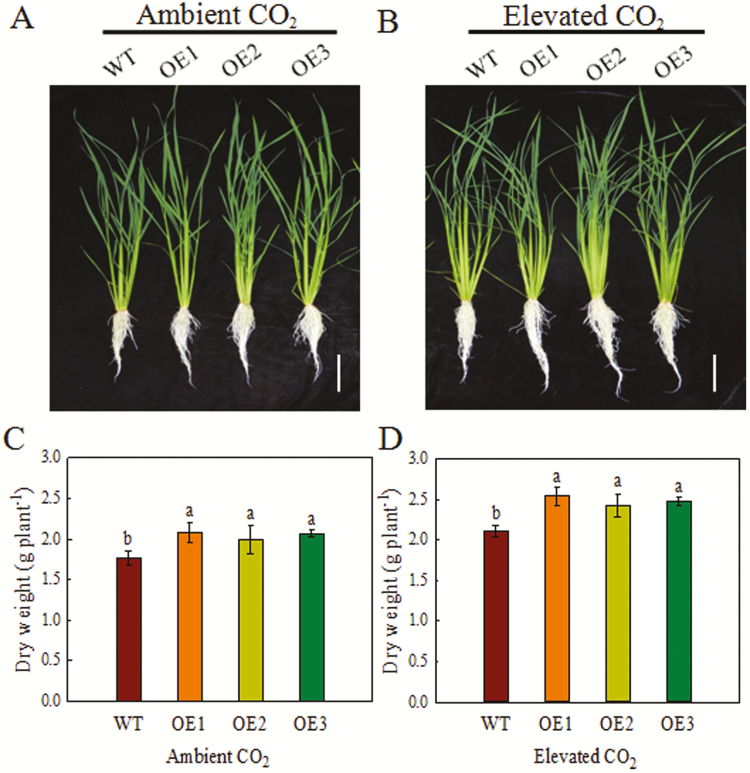
Growth of rice plants under ambient and elevated [CO_2_]. Seedlings were grown under ambient [CO_2_] (400 ppm) or elevated [CO_2_] (800 ppm) in growth chambers for 4 weeks. (A, B) Growth phenotypes of the OE lines and the WT under ambient [CO_2_] (400 ppm) or elevated [CO_2_] (800 ppm) for 4 weeks. Scale bar: 10 cm. (C, D) Total dry weight of the OE lines and the WT grown under ambient [CO_2_] or elevated [CO_2_] for 4 weeks. Values are means ±SD (*n*=5); different letters indicate significant differences (*P*<0.05).

We determined the growth of the OE lines and the WT under ambient and elevated [CO_2_] over 4 weeks (Fig. 4). Compared with ambient [CO_2_], the total dry weight of the OE lines and the WT were significantly higher in the presence of an elevated [CO_2_]. Moreover, the total dry weight of the OE lines was 13–18% and 15–20% higher than that of the WT under ambient and elevated [CO_2_], respectively (Fig. 4C, D).

### Carbohydrate content and sucrose transport under ambient and elevated [CO_2_]

**Fig. 5.**
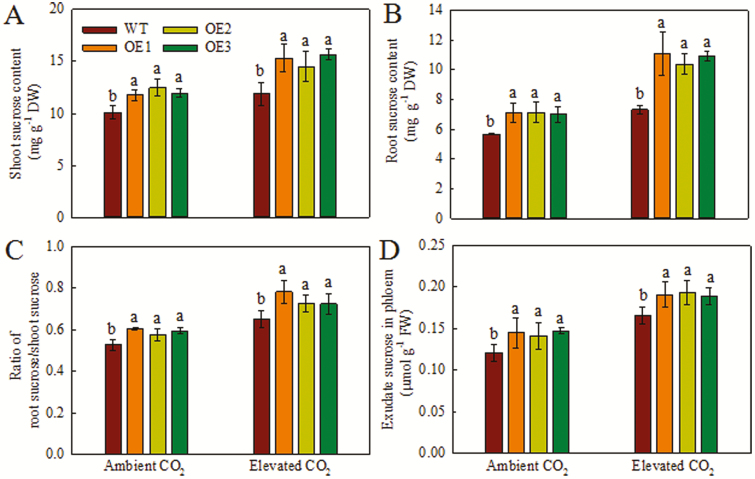
Effect of *OsPIP1;2* overexpression on phloem sucrose transport in rice plants. (A, B) Sucrose contents of the shoot (A) and root (B) of the OE lines and the WT grown under ambient [CO_2_] (400 ppm) or elevated [CO_2_] (800 ppm) in growth chambers for 4 weeks. (C) Ratio of root sucrose/shoot sucrose in the OE lines and the WT grown under ambient [CO_2_] or elevated [CO_2_] for 4 weeks. (D) Phloem sucrose contents of the OE lines and the WT grown under ambient [CO_2_] or elevated [CO_2_] for 4 weeks. Values are means ±SD (*n*=5); different letters indicate significant differences (*P*<0.05).

Phloem sucrose transport and sucrose content were measured in the WT and OE lines under ambient and elevated [CO_2_] (Fig. 5). The shoot sucrose content, root sucrose content, ratio of root/shoot sucrose, and phloem sucrose transport activity in the OE lines were 10–18%, 17–20%, 10–15%, and 18–21% higher, respectively, than those of the WT under ambient [CO_2_]. Under elevated [CO_2_], the shoot sucrose content, root sucrose content, and ratio of root/shoot sucrose of the OE lines were 19–28%, 24–34%, 11–20%, and 13–16%, respectively, higher than those of the WT. Phloem sucrose transport activity in the OE lines was 13–16% higher than that in the WT (Fig. 5), indicating that the OE lines allocate more carbon from source to sink. Therefore, we investigated phloem sucrose transport from shoot (source) to panicle (sink). The panicle sucrose content of the T4-, T5-, and T6-generation OE lines was 41–49%, 51–66%, and 37–50% higher than that of the WT, respectively (Table 2); this may influence the rice yield.

**Table 2.** Comparison of agronomic traits and panicle sucrose content of rice plants grown in paddy field in 2016 and 2017

	Effective tiller No. per plant	Spikelets per panicle	Panicle sucrose content (mg g−1)
2016 Nanjing			
WT	18.7 ± 0.9b	85 ± 8.4b	10.2 ± 0.3b
OE1	22.3 ± 1.4a	97 ± 11a	14.4 ± 0.5a
OE2	24.5 ± 0.7a	101 ± 14a	14.8 ± 1.1a
OE3	21.9 ± 1.5a	95 ± 8.8a	15.2 ± 1.5a
2017 Sanya			
WT	21.3 ± 0.5b	79 ± 8.3b	9.5 ± 0.8b
OE1	27.7 ± 1.2a	95 ± 4.1a	14.3 ± 1.5a
OE2	25.3 ± 1.6a	97 ± 8.2a	15.8 ± 1.2a
OE3	25.9 ± 1.7a	92 ± 5.8a	15.2 ± 0.5a
2017 Nanjing			
WT	15.0 ± 2.4b	88 ± 6.5b	11.5 ± 0.8b
OE1	21.0 ± 1.7a	98 ± 7.3a	15.7 ± 0.6a
OE2	17.7 ± 3.1a	95 ± 6.7a	17.3 ± 1.2a
OE3	18.9 ± 1.6a	96 ± 7.2a	16.1 ± 0.9a

Statistical analysis of data is from T4–T6 generations. Values are means ±SD (*n*=12). Different letters indicate significant differences at the *P*<0.05 level in rice plants.

### Alteration of *OsPIP1;2* expression affects rice grain yield

**Fig. 6.**
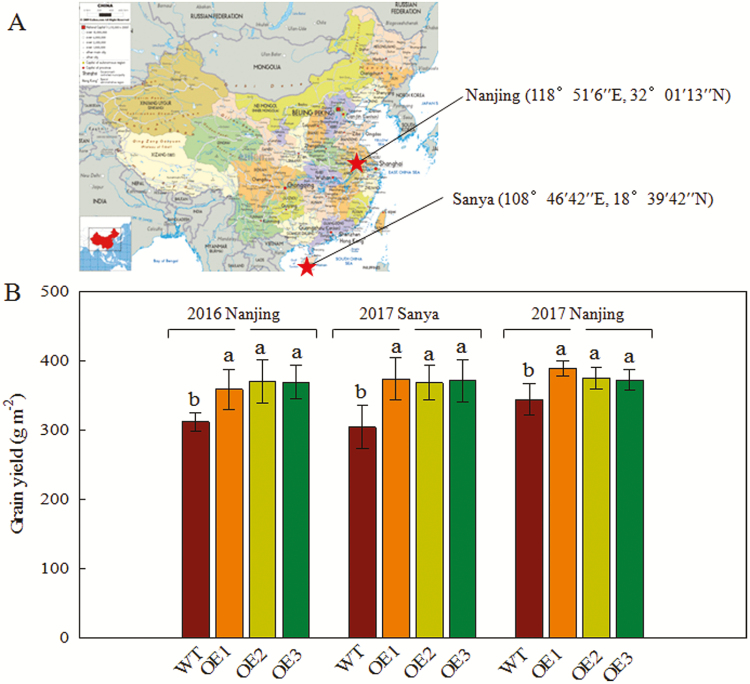
Grain yield of transgenic rice plants grown in a paddy field in 2016 and 2017. (A) The locations of the field experiments in 2016 (Nanjing) and 2017 (Nanjing and Sanya). (B) Grain yield of transgenic rice plants. Values are means ±SD (*n*=3); different letters indicate significant differences (*P*<0.05).

To examine the influence of *OsPIP1;2* on rice grain yield, *OsPIP1;2* WT and OE lines were cultivated in a field (Fig. 6; Table 2). The agricultural traits of the T4–T6 generations of the OE lines and the WT were investigated at Nanjing City, Jiangsu Province and Sanya City, Hainan Province from 2016 to 2017 (Table 2). The effective tiller number and spikelets per panicle of the OE lines were 17–40% and 12–23% higher than those of the WT. The grain yield of the OE lines was enhanced by 13–25% (T4 generation) at Nanjing, by 18–23% (T5 generation) at Sanya, and by 13–36% (T6 generation) at Nanjing, relative to the WT (Fig. 6). Therefore, overexpression of *OsPIP1;2* enhances rice yield in the field.

## Discussion

We report here that overexpression of *OsPIP1;2* enhances the net CO_2_ assimilation rate by improving CO_2_ diffusion in the leaf, which increases the growth and yield of rice plants. Rice *OsPIP1;2* belongs to the PIP1 family, among which PIP1;2 had a function in CO_2_ diffusion in tobacco and Arabidopsis (Uehlein *et al.*, 2003; Heckwolf *et al.*, 2011; Sade *et al.*, 2014). We reported previously that oocytes transfected with *OsPIP1;2* did not show water-transport activity (Ding *et al.*, 2016), suggesting that water transport activity was not enhanced in the OE lines. Interestingly, the amino acid sequences of OsPIP1;2 and other aquaporins related to CO_2_ permeability are highly conserved (Supplementary Fig. S2). According to Mori *et al.* (2014), OsPIP1;2 had the same amino acid residue at the C-terminal end of the E-loop as barley aquaporin, which was permeable to CO_2_ in the *Xenopus laevis* oocyte expression system. In addition, the relative expression of *OsPIP1;2* was significantly increased under elevated [CO_2_] (Fig. 1). Therefore, OsPIP1;2 may be associated with CO_2_ permeability. In addition, *g*_m_ related closely to the modification of aquaporins in tobacco and Arabidopsis (Uehlein *et al.*, 2003; Heckwolf *et al.*, 2011; Sade *et al.*, 2014). In our study, *OsPIP1;2* expression was significantly up-regulated in transgenic plants relative to the WT (Fig. 2). The *OsPIP1;2*-OE transgenic rice lines had higher *g*_m_ and *A*_net_, and the *g*_m_ of OE lines was 1.5-fold higher than that of the WT (Fig. 3D; Table 1). Our results are consistent with the observation in NtAQP1 (NtAQP1;2) OE tobacco plants (Uehlein *et al.*, 2003). Although, *g*_m_ and *g*_s_ are in general correlated (Lauteri *et al.*, 1997; Loreto *et al.*, 2003), the *g*_s_ of the OE lines was observed to be only 0.3-fold higher than that of the WT (Supplementary Fig. S4), indicating that a high *A*_net_ is mainly contributed by increased *g*_m_. The cholorplastic CO_2_ concentration was also higher in the OE lines than in the WT (Table 1), indicating that OsPIP1;2 influences mesophyll CO_2_ conductance. In general, high *g*_s_ usually causes lower WUE (Lawson and Blatt, 2014). However, in our study, the *OsPIP1;2* OE lines did not show decreased WUE even though the *g*_s_ increased. The reason is the enhanced net rate of CO_2_ assimilation in overexpression of *OsPIP1;2*, which showed high *g*_m_ (Supplementary Figs S4 and S5). Therefore, a potential approach for crop plants is to increase *g*_s_, maintaining WUE without substantial cost in overexpression of *OsPIP1;2*.

*g*_s_, but not stomatal density or size, differed between the OE lines and the WT (Fig. 3; Supplementary Fig. S4; Supplementary Table S5). These results are partly consistent with the findings of Hanba *et al.* (2004), who reported that overexpression of barley aquaporin *HvPIP2;1* in rice increased *g*_s_ by 27%. The *A*_net_–*C*_i_ curves revealed that the response to increasing *C*_i_ was enhanced during the first phase in the OE lines (Fig. 3A), consistent with the reduced *C*_i_ in *atpip1;2* lines reported by Heckwolf *et al.* (2011). According to the *A*_net_–*C*_i_ curves, *V*_cmax_ and CO_2_ assimilation rate of the OE lines were increased (Table 1), but this is not due to the difference in leaf N content (Supplementary Fig. S7). In addition, under the FACE system, *V*_cmax_ decreases as an acclimatory response to long-term elevated [CO_2_] (Ainsworth and Long, 2005). In the present study, *g*_m_ and *V*_cmax_ were increased in OE lines, which suggested that *g*_m_ enhancement may increase *V*_cmax_ as the early response to CO_2_ in rice plants.

Sucrose is the major translocated photosynthetic product and the main form of carbon on which plant sinks grow (Sung *et al.*, 1989). In this study, the shoot sucrose content of the OE lines was higher than that of the WT (Fig. 5A). Sucrose acts as a signaling molecule and provides energy for root growth and development (Chiou and Bush, 1998). Efficient sucrose transport from shoot to root via phloem plays a key role in root growth (Salerno and Curatti, 2003). Plant acclimation to elevated [CO_2_] has been associated with an increase in carbohydrate content (Weigel and Manderscheid, 2012). In the present study, the OE lines had higher biomass than the WT under both ambient and elevated [CO_2_] (Fig. 4), consistent with the results of Kim *et al.* (2001). This is because the OE lines showed higher carbohydrate content than the WT (Fig. 5). Our results suggest that *OsPIP1;2* is involved in the response to elevated [CO_2_] and increases the root/shoot sucrose concentration ratio (Figs 4 and 5). Although the root/shoot sucrose concentration ratio is not a good proxy for phloem export, it is also affected by the sucrose consumption rates in organs. Thus, we examined phloem transport in rice plants. Under ambient [CO_2_], the OE lines showed a higher root sucrose content than the WT (Fig. 5D). Therefore, the OE lines exhibited higher phloem sucrose transport activity compared with the WT, suggesting greater carbon allocation from source to sink (Fig. 5D).

Sucrose is an important determinant of the number of grains in rice spikelets, and an increased number of spikelets per panicle is key for enhancing grain yield (Kato *et al.*, 2007). In the field experiment, the panicle sucrose content of the OE lines was markedly higher (Table 2), suggesting greater sucrose export from leaves to seeds, relative to the WT. This suggests that the OE lines were source limited, but not sink limited, in grain yield increase. Further, the OE lines had a larger number of spikelets per panicle than the WT (Table 2), which may be due to their greater sink capacity. This finding is consistent with prior reports that the yield potential of rice is enhanced by its large sink capacity, itself related to the large number of spikelets per panicle (Peng *et al.*, 2008). Therefore, overexpression of *OsPIP1;2* increased the number of spikelets per panicle in rice plants by enhancing sucrose transport from leaf to panicle by increasing the net CO_2_ assimilation rate. This likely contributed to the increased yield of the OE lines in the field experiment (Fig. 6).

In addition, the leaf relative water content and dry mass per unit area reflect the water status of the plant (Jones, 2007). The leaf relative water content and dry mass per unit area did not differ between the OE lines and the WT (Supplementary Table S4), indicating that water transport in leaves was unaffected by overexpression of *OsPIP1;2*. The number of leaves per plant also did not differ between the OE lines and the WT (Supplementary Table S4), suggesting that the OE lines have the same developmental rates. Our results indicate that the effect of *OsPIP1;2* on rice growth and yield is largely due to the facilitation of CO_2_ transport rather than the modulation of water transport and developmental rate. In conclusion, our results indicate that overexpression of *OsPIP1;2* modulates the number of spikelets per panicle by increasing leaf CO_2_ diffusion, photosynthetic performance, and phloem sucrose transport; together, these effects have a positive effect on rice yield.

## Supplementary data

Supplementary data are available at *JXB* online.

Fig. S1. Daytime respiration rate (*R*_d_) of the transgenic rice plants.

Fig. S2. Homology modeling of OsPIP1;2.

Fig. S3. Net rate of CO_2_ assimilation (*A*_net_) to PPFD of rice plants under 400 ppm CO_2_.

Fig. S4. Stomatal conductance (*g*_s_) of rice plants under 400 ppm CO_2_.

Fig. S5. Water-use efficiency of rice plants under 400 ppm CO_2_.

Fig. S6. Chlorophyll content and net rate of CO_2_ assimilation (*A*_net_, μmol m^−2^ s^−1^) of newly and fully expanded leaves of rice plants at the flowering stage (Flowering), middle grain-filling stage (Mid-Fill), and end grain-filling stage (End-Fill).

Fig. S7. Leaf N content of rice plants under 400 ppm CO_2_.

Table S1. Primers used for construction of vectors.

Table S2. Primers used for RT-PCR analysis.

Table S3. Primers used for real-time quantitative PCR analysis.

Table S4. Morphological and physiological parameters of the rice plants.

Table S5. Stomatal size in leaves of the transgenic rice plants.

## Supplementary Material

Supplementary Table S1-S5

Supplementary Figure S1-S7
