# Erratum: Overexpression of rice aquaporin *OsPIP1;2* improves yield by enhancing mesophyll CO2 conductance and phloem sucrose transport

**DOI:** 10.1093/jxb/erz043

**Published:** 2019-03-14

**Authors:** Feiyun Xu, Ke Wang, Wei Yuan, Weifeng Xu, Shuang Liu, Herbert J Kronzucker, Guanglei Chen, Rui Miao, Maoxing Zhang, Ming Ding, Liang Xiao, Lei Kai, Jianhua Zhang, Yiyong Zhu

**Affiliations:** 1 Jiangsu Collaborative Innovation Center for Solid Organic Waste Resource Utilization, College of Resources and Environmental Science, Nanjing Agricultural University, Nanjing, China; 2 College of Life Sciences and Joint International Research Laboratory of Water and Nutrient in Crops, Fujian Agriculture and Forestry University, Fuzhou, China; 3 School of Agriculture and Food, Faculty of Veterinary and Agricultural Sciences, The University of Melbourne, Australia; 4 The Key Laboratory of Biotechnology for Medicinal Plants of Jiangsu Province, Jiangsu Key Laboratory of Phylogenomics and Comparative Genomics, School of Life Sciences, Jiangsu Normal University, Xuzhou, China; 5 Department of Biology, Hong Kong Baptist University, and the State Key Laboratory of Agrobiotechnology, Chinese University of Hong Kong, Hong Kong, China

*Journal of Experimental Botany*, Volume 70, Issue 2, 7 January 2019, Pages 671–681, https://doi.org/10.1093/jxb/ery386

In this paper author Ke Wang was not listed as a joint first author and the name of author Shuang Liu was reversed. These errors have been corrected online.

