# Supplementary material for: Overexpression of rice aquaporin OsPIP1;2 improves yield by enhancing mesophyll CO2 conductance and phloem sucrose transport
Source: J Exp Bot. 2018 Dec 11;70(2):671–81. doi: 10.1093/jxb/ery386 (PMC6322580; doi:10.1093/jxb/ery386)
Supplement: Supplementary Table S1-S5 [file ery386_supplementary-table-s1-s5.pdf]

Overexpression of aquaporin *OsPIP1;2* in rice improves yield by enhancing mesophyll CO_2 conductance and phloem sucrose transport

Table S1. Primers used for construction of vectors.

Primer name		Primer (5′-3′)
OsPIP1;2-OE	FP1	CAGTGGTCTCNTTTGATGGGGAAGGACGAGGTGAT
	RP1	CAGTGGTCTCNACCGGGACATGTGAGTCGCG
	FP2	CAGTGGTCTCNCGGTCTTGGCGCCGCTGCCAATCG
	RP2	CAGTGGTCTCNATGGTTGTGCCACGCCTTCT
	FP3	CAGTGGTCTCNCCATTGGATCTTCTGGGTCGGCCC
	RP3	CAGTGGTCTCNAGAGTCACGCGTTGCTCCTGAAGG
OsPIP1;2 promoter*::GUS*	FP	CCGTCGACTCTCTTCCGTCAGTCTCAGC
	RP	GGGGTACCCTCTCTACTTTCTCTCTACT
35S::OsPIP1;2::GFP	FP	CTAAGCTTTGGAGGGGAAGGAGGAGGA
	RP	CACTGCAGTTACGACCTGCTCTTGAATG

Table S2. Primers used for RT-PCR analysis

Gene name (DNA Accession No.)	Forward primer (5′-3′)	Reverse primer (5′-3′)
OsActin (AB047313)	GGAACTGGTATGGTCAAGGC	AGTCTCATGGATAACCGCAG
OsPIP1;2 (AK098849)	CGACGACAAGGACTACAAGGAG	CAGAAGATCCAATGGTCATCCC

Table S3. Primers used for real-time quantitative PCR analysis

Gene name (DNA Accession No.)	Forward primer (5′-3′)	Reverse primer (5′-3′)
OsActin (AB047313)	GGGTTCACAAGTCTGCCTATTGT	ACGGGACACGACCAAGGA
OsPIP1;2 (AK098849)	GACCATTGGATCTTCTGGGT	TACGACCTGCTCTTGAATGG

Table S4 Morphological and physiological parameters of the rice plants

Genotype	WT	OE1	OE2	OE3
Total Chl. content (g m^{-2})	0.31±0.02a	0.32±0.02a	0.30±0.01a	0.32±0.04a
Chl.a (g m^{-2})	0.25±0.01a	0.25±0.02a	0.23±0.01a	0.26±0.01a
Chl.b (g m^{-2})	0.06±0.02a	0.07±0.09a	0.07±0.03a	0.06±0.07a
Chl.a/b ratio	4.17±0.33a	3.58±0.36a	3.29±0.24a	4.33±0.32a
Number of leaves per plant	26.76±2.31a	28.63±2.54a	29.41±3.27a	27.72±3.19a
Relative water content (%)	80.77±8.52a	76.62±3.58a	77.29±1.46a	75.80±2.53a
Leaf dry mass per unit area(g m^{-2})	25.78±3.26a	24.78±1.94a	26.54±2.45a	27.35±1.41a

Note: Newly and fully expanded leaves grown in the chamber under ambient [CO_2] for 4 weeks were used for measurement. Values are means ± SDs (n = 3). Different letters indicate significant differences at the $P < 0.05$ level in rice plants.

Table S5 Stomatal size in leaves of the transgenic rice plants.

Stomatal size (μm)	WT	OE1	OE2	OE3
Adaxial surface	22.4 ±1.7a	21.9 ±1.9a	24.7 ±1.9a	20.3 ±1.8a
Abaxial surface	23.1 ±1.4a	21.9 ±1.6a	23 ±1.5a	21.1 ±1.7a

Note: Newly and fully expanded leaves grown in the chamber under ambient [CO_2] for 4 weeks were used for stomatal measurement. Values are means ± SDs (n = 30). Different letters indicate significant differences at the $P < 0.05$ level in rice plants.
