# Supplementary material for: Overexpression of rice aquaporin OsPIP1;2 improves yield by enhancing mesophyll CO2 conductance and phloem sucrose transport
Source: J Exp Bot. 2018 Dec 11;70(2):671–81. doi: 10.1093/jxb/ery386 (PMC6322580; doi:10.1093/jxb/ery386)
Supplement: Supplementary Figure S1-S7 [file ery386_supplementary-figure-s1-s7.pdf]

Overexpression of aquaporin *OsPIP1;2* in rice improves yield by enhancing mesophyll CO_2 conductance and phloem sucrose transport

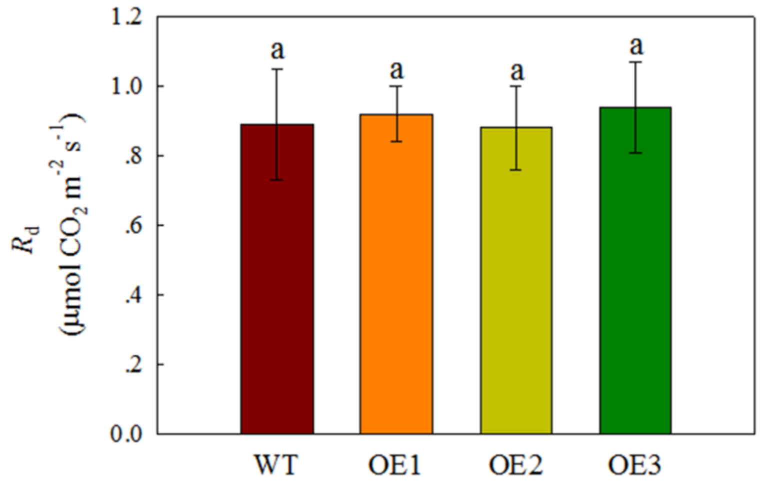

Fig. S1 Daytime respiration rate (R_d) of the transgenic rice plants. Rice plants were grown in the chamber under ambient [CO_2] for 4 weeks. Values are means ±SDs (n = 5). Different letters indicate significant differences at the $P < 0.05$ level in rice plants.

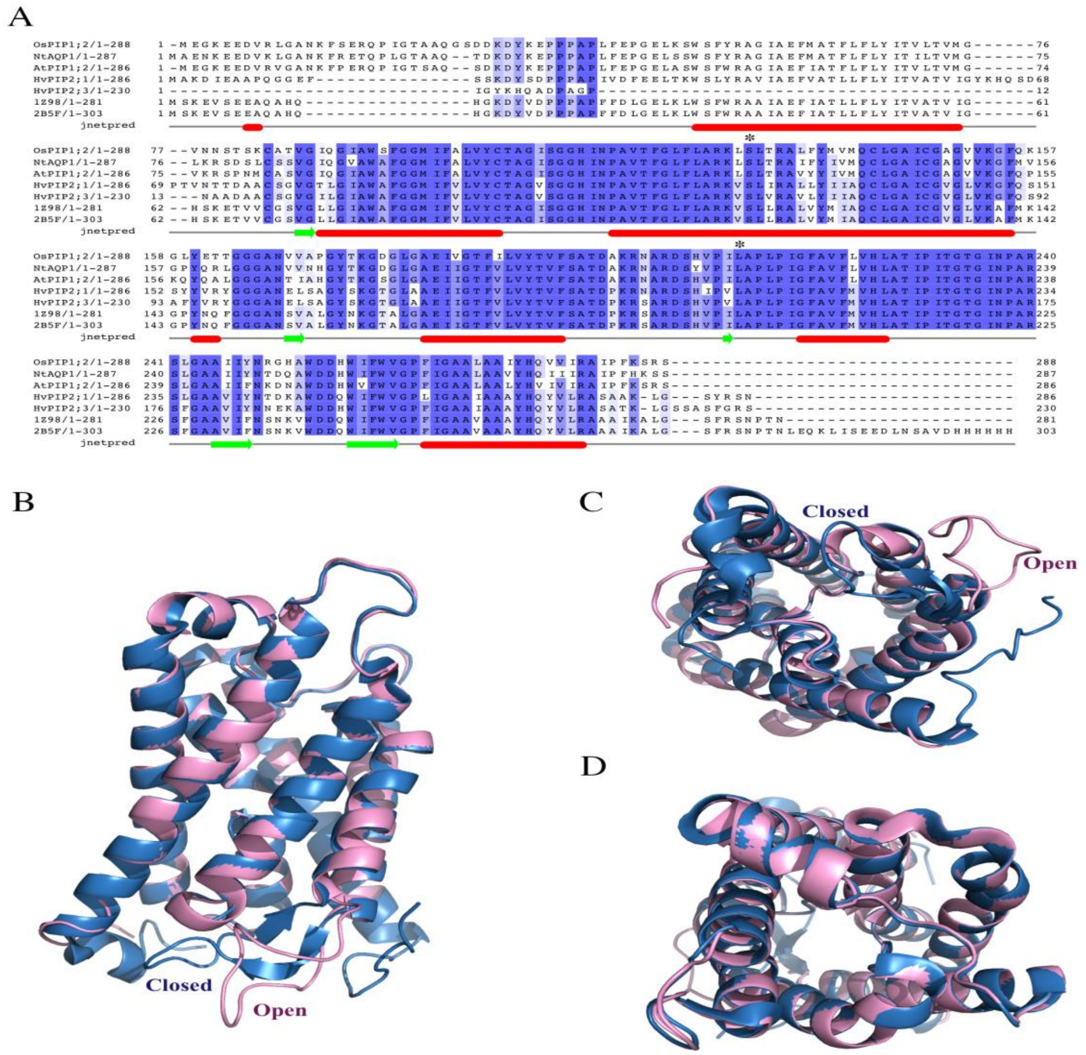

Fig. S2. Homology modeling of OsPIP1;2. (A) Structure-based sequence alignment of OsPIP1;2 with NtAQP1, AtPIP1;2, HvPIP2;1 and HvPIP2;3. Identical amino acid residues are shown on a blue background and dashes indicate gaps. (B-D) Homology model of OsPIP1;2 was constructed using the SoPIP2;1 (PDB code: 3JW8) structure as template.

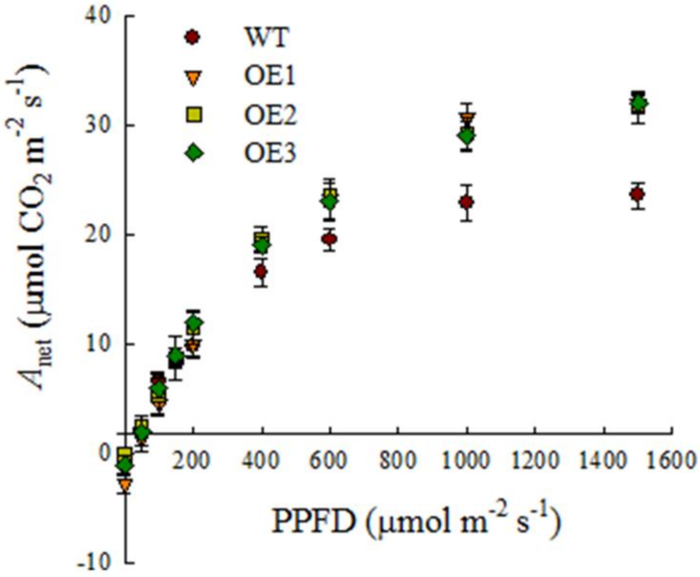

Fig. S3. Net rate of CO_2 assimilation (A_{net}) to PPFD of rice plants under 400 ppm CO_2. Rice plants were grown in the chamber under ambient [CO_2] for 4 weeks. Measurements were conducted at 400 μmol m^{-2} s^{-1} CO_2. The temperature of the leaf chamber was maintained at 25 ℃. The relative humidity was kept at 50-60% in the leaf chamber. Values are means ±SDs (n = 5).

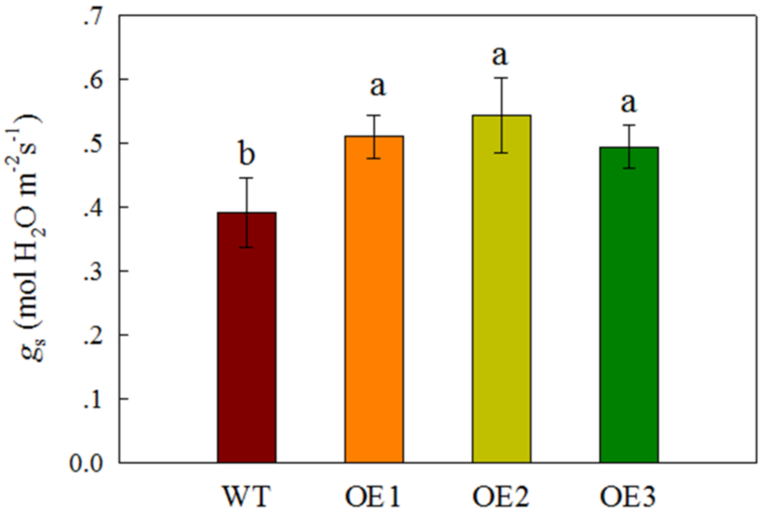

Fig. S4 Stomatal conductance (g_s) of rice plants under 400 ppm CO_2. Rice plants were grown in the chamber under ambient [CO_2] for 4 weeks. Measurements were conducted at 400 μmol m^{-2} s^{-1} CO_2. The temperature of the leaf chamber was maintained at 25 ℃, with photosynthetic active photon flux density (PPFD) of 1500 μmol m^{-2} s^{-1}. The relative humidity was kept at 50-60% in the leaf chamber. Values are means ± SDs (n = 5). Different letters indicate significant differences at the $P < 0.05$ level in rice plants.

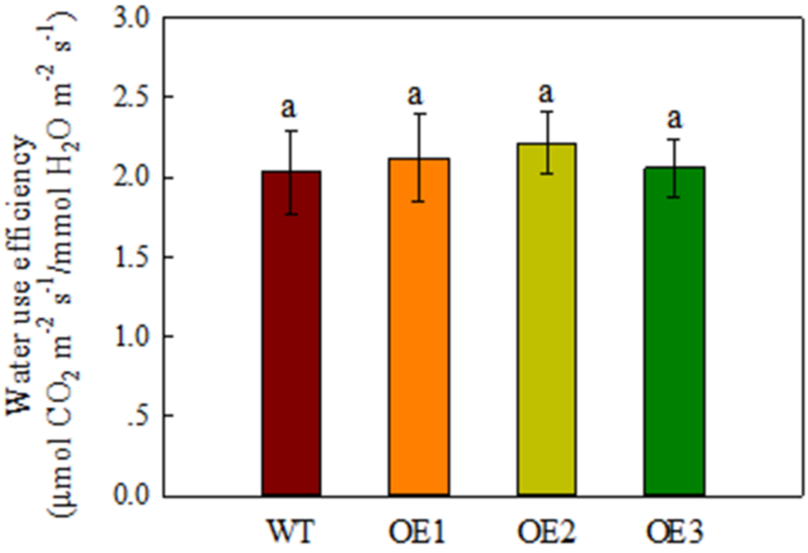

Fig. S5 Water use efficiency of rice plants under 400 ppm CO_2. Rice plants were grown in the chamber under ambient [CO_2] for 4 weeks. Measurements were conducted at 400 μmol m^{-2} s^{-1} CO_2. The temperature of the leaf chamber was maintained at 25 ℃, with photosynthetic active photon flux density (PPFD) of 1500 μmol m^{-2} s^{-1}. The relative humidity was kept at 50-60% in the leaf chamber. Water us efficiency was calculated as the ratio between net rate of CO_2 assimilation and transpiration rate. Values are means ±SDs (n = 5).

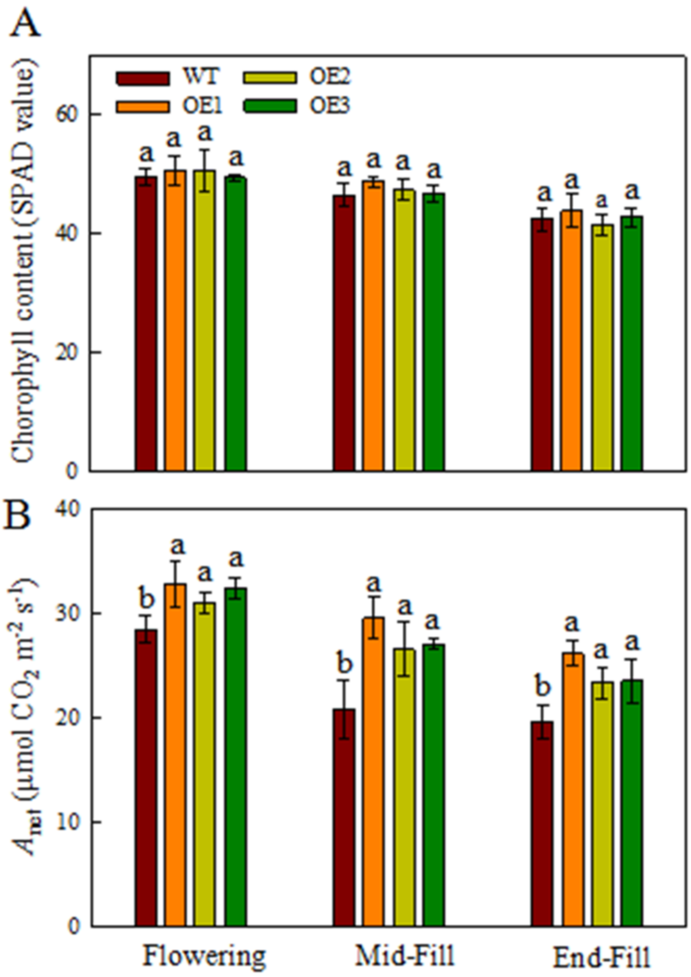

Fig. S6. Chlorophyll content (A) and net rate of CO_2 assimilation (A_{net}, μmol m^{-2} s^{-1}) (B) of newly and fully expanded leaves of rice plants at the flowering stage (Flowering), middle grain filling stage (Mid-Fill) and end grain filling stage (End-Fill). Seedlings were grown in paddy soil in Nanjing Agricultural University in 2016. Measurements were conducted at 400 μmol m^{-2} s^{-1} CO_2. The temperature of the leaf chamber was maintained at 25 ℃, with photosynthetic active photon flux density (PPFD) of 1,500 μmol m^{-2} s^{-1}. The relative humidity was kept at 50-60% in the leaf chamber. Values are means ± SDs (n = 12). Different letters indicate significant differences at the $P < 0.05$ level in rice plants.

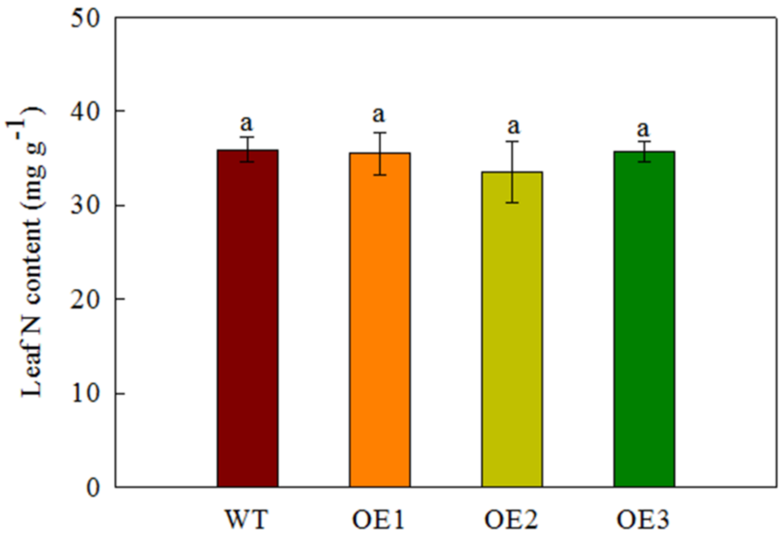

Fig. S7 Leaf N content of rice plants under 400 ppm CO_2. Rice plants were grown in the chamber under ambient [CO_2] for 4 weeks. Values are means ±SDs (n = 5). Different letters indicate significant differences at the $P < 0.05$ level in rice plants.
